# Two Mischievous Bills

**Published:** 1891-04

**Authors:** 


### BUFFALO MEDICAL AND SURGICAL JOURNAL

A MONTHLY REVIEW OF MEDICINE AND SURGERY.

Vol. XXX. APRIL, 1891. No. 9.

*EDITORS:*

THOMAS LOTHROP, M. D. - - WM. WARREN POTTER, M. D.

All communications, whether of a literary or business character, should be addressed to the editors : 284 Franklin Street, Buffalo, N. Y.

### Editorial.

### TWO MISCHIEVOUS BILLS.

There are two bills now pending in the Legislature of the State of New York which, if they should become laws, will serve to effectually block the wheels of progress in regard to reforms in medical education. The first, Senate bill No. 346, was introduced by Mr. Jacobs, February 9, 1891, and is as follows :

Section 1. Section eleven of chapter five hundred and seven of the laws of eighteen hundred and ninety, is hereby amended so as to read as follows :

Sec. 11. This act shall not apply to any student who duly matriculated in some legally incorporated medical college before the fifth day of June, eighteen hundred and ninety, or who began the study of medicine with some legally authorized physician prior to the fifth day of June, eighteen hundred and eighty-nine, provided that such student within three months after the enactment of this amendment shall file with the Secretary of the Board of Regents of the University of the State of New York, a certificate setting forth the fact of such matriculation or the beginninng of such study, verified by the applicant ; and signed by the secretary of the faculty of the college in which he matriculated, or accompanied by the affidavit of the physician under whom he began the study of medicine, setting forth the time when such student began such study.

Sec. 2. This act shall take effect immediately.

Should this bill pass, it will practically nullify or make nugatory the provisions of the present State Medical Examiners Law for the next two years at least. It appears innocent, but is fraught with danger and should be defeated.

The second is known as Senate Bill No. 408, and was introduced by Mr. Collins, March 9, 1891. A similiar bill was simultaneously introduced in the Assembly by Mr. Green. This bill practically creates a new system for the organization of a Medical Examining Board, in that it confers upon the faculties of Cornell University, Columbia College, Union University, the University of the City of New York, the University of Syracuse, the University of Rochester, Niagara University, and Colgate University, the power to nominate an Examining Board to consist of fourteen members. The underlying features of the present law are such that a complete divorcement of the teaching and licensing powers shall be wrought, which is the only safe basis for the construction of a State Medical Examining Board. The bill under consideration restores the licensing power to the faculties, and complicates unnecessarily the system which has heretofore prevailed. It subjects the new graduate to the expense of obtaining a State license, of a kind practically no better than the diploma that he has already procured from the faculty of his Alma Mater. If the State compels him to undergo the second examination, and charges him a fee therefor, should it not give him an equivalent for his expenditure of time and money ?

There are many subtle and apparently innocent provisions of this bill that will bear careful study and analysis, but we have not the space now at command to devote to this important work. We advise those interested to procure a copy and weigh its features considerately.

The Medical Society of the County of Erie, in special session March 20, 1891, paid respect to it in debate, and afterward unanimously passed the following preamble and resolution offered by Dr. Thos. Lothop, vice-president of the faculty of Niagara University :

WHEREAS, The Medical Society of the County of Erie has for many years earnestly contended for a State Licensing Board for physicians ; and

WHEREAS, The membership of this Society includes the members of the Medical Faculty of the University of Buffalo and the Medical Faculty of Niagara University ; and

WHEREAS, This Society has practically been without division upon the question of having a State Licensing Board, and also upon the principle that such board should be composed of men not engaged in the work of teaching medicine; and

WHEREAS, After years of study and consideration the Legislature of last year did enact a law providing for a State license for physicians, the same to take effect September 1st of this year; and

WHEREAS, Certain bills are now pending at Albany known as Senate Bill No. 346 and Senate Bill No. 408, which have for their object and purpose postponement of the operation of the law creating the State license for physicians and the further object of placing the said licensing of physicians in the hands of certain medical schools; and in other ways changing the methods of State examination and State license in such wise as to destroy their prospect for usefulness, to the injury of the medical profession and to the prejudice of the public health; therefore

*Resolved*, That the Medical Society of the County of Erie, for itself and on behalf of the Medical Faculties of the University of Buffalo and Niagara University, unreservedly disapproves of Senate Bills No. 346 and No. 408, and urges upon our Members of the Legislature to use all honorable means to prevent their passage.

JNO. A. PETTIT, *President pro tem.*

ELI H. LONG, *Secretary.*

It thus appears that the bills introduced in the Senate by Mr. Collins and in the Assembly by Mr. Green, were drawn without the consent of the faculty of Niagara University, and it may be that some of the other Colleges or Universities named in the bill have been made to bear unwilling testimony of their own disgrace. If so, it behooves them to make all haste to purge themselves as thoroughly of the very suspicion of evil as Niagara University has done with such commendable promptitude.

